# Long-term economc burden and related influencing factors of pediatric cataracst: A population-based study in South Korea

**DOI:** 10.1371/journal.pone.0328781

**Published:** 2025-08-21

**Authors:** Semin Jang, Sang Jun Park, Seo Yoon Choi, Migyeong Jeong, Siin Kim, Hae Sun Suh

**Affiliations:** 1 Department of Regulatory Science, Graduate School, Kyung Hee University, Seoul, Republic of Korea; 2 Institute of Regulatory Innovation through Science (IRIS), Kyung Hee University, Seoul, Republic of Korea; 3 College of Pharmacy, The University of Texas at Austin, Austin, Texas, United States of America; 4 Department of Ophthalmology, Seoul National University Bundang Hospital and College of Medicine, Seoul National University, Seongnam, Republic of Korea; 5 College of Pharmacy, Kyungsung University, Busan, Republic of Korea; 6 College of Pharmacy, Kyung Hee University, Seoul, Republic of Korea; Alexandria University Faculty of Medicine, EGYPT

## Abstract

Pediatric cataracts are a significant cause of childhood blindness, requiring long-term management. This study evaluated the economic burden of pediatric cataract surgery in South Korea and identified factors influencing the medical costs, particularly in patients with complications after cataract surgery. We used National Health Insurance Service claims data, covering the Korean population, including pediatric patients (aged ≤5 years) who underwent cataract surgery between 2004 and 2011. We measured healthcare resource utilization (outpatient, inpatient, and emergency department visits) and cataract-related medical costs over an 8-year follow-up after cataract surgery. We further explored factors influencing medical costs using a generalized linear model. A total of 1297 pediatric patients (mean age: 2.2 years, 56.8% male) were included. Over 8 years, patients had an average of 30 outpatient visits, 2.7 inpatient visits, and 3 emergency department visits. The total average medical cost over 8 years was $2,391 per patient, with significantly higher costs in those developed retinal detachment ($7729) or glaucoma ($8,821) developed after the surgery. Additionally, higher costs were associated with older age at surgery, higher income, the presence of postoperative complications, and multiple surgeries. Pediatric cataract surgery resulted in a considerable long-term economic burden, particularly when accompanied by postoperative complications. This study provides real-world evidence underscoring the need for improvement in pediatric cataract management to ensure optimal care for young patients at risk of vision impairment.

## Introduction

Cataracts, characterized by the clouding of the eye’s lens, can develop at any age and may lead to vision impairment. In different to senile cataracts, pediatric cataracts represent a significant cause of treatable childhood blindness, accounting for 7–20% of such cases [1–3]. Unlike adults, where vision is corrected immediately after the surgery, children require timely and appropriate treatment during the critical vision development stages, involving surgery and long-term ophthalmological management, including amblyopia treatment, for normal vision development [4,5]. Occurring during crucial visual development stages in children, pediatric cataracts can lead to permanent vision impairment by causing amblyopia [6–8]. The prevalence of pediatric cataracts ranges from 2.2 to 13.6 per 10,000 children; however, early treatment can effectively prevent blindness [6,9,10].

For cataracts, vision improvement options include glasses, contact lenses, and eye drops to slow progression. However, surgery remains the definitive treatment as these methods cannot clear a clouded lens [4]. Pediatric cataract surgery presents additional challenges such as the need for general anesthesia, longer surgery times, caregiver involvement, a high rate of postoperative complications, and ongoing vision care after surgery. These factors contribute to an increased ophthalmological burden and significant long-term social costs [11–13].

There is a paucity of nationwide data addressing the burden of pediatric cataracts, and therefore, healthcare policies for pediatric cataracts remain underdeveloped. Previous studies on the costs of pediatric cataract surgery relied on hospital data, featuring relatively small sample sizes and focusing on cross-sectional rather than longitudinal analyses [11–13]. Furthermore, to our knowledge, no studies have reported the costs incurred following cataract surgery over a long-term follow-up period, nor the expenses associated with complications after the surgery [12–17]. Therefore, a nationwide population-based study focusing on pediatric cataracts and detailing the medical costs incurred following cataract surgery was required.

This study aimed to analyze the medical costs incurred by pediatric patients who underwent cataract surgery under 5 years of age, following cataract surgery, including those related to postoperative complications, and to identify the factors influencing these costs. The findings could help develop a more comprehensive treatment system for pediatric cataracts.

## Methods

### Data source

In this retrospective longitudinal study, we utilized national claims data from the National Health Insurance Service (NHIS) in South Korea. The NHIS claims data (NHIS-2021-1-140), a de-identified nationwide database, covers the entire South Korean population of 50 million. We screened the data from January 1, 2002, to December 31, 2019, comprising 1,862,725 patients who underwent cataract surgery between January 1, 2004, and December 31, 2011, from the entire population. It includes medical claims information on healthcare utilization, examinations, sociodemographic characteristics, disease diagnoses, procedure records, drug prescriptions, and death records [18]. Researchers had access to the data between April 23, 2019, and April 23, 2023. All data were de-identified, thus, the authors could not identify individual participants.

### Eligible criteria and study design

We included the data of pediatric patients with cataracts (aged ≤ 5 years) who underwent cataract surgery at an ophthalmology department between 2004 and 2011. The cataract surgeries were identified using the procedure code. We excluded patients without a recorded birth year or claims number, those who underwent their first cataract surgery between 2012 and 2019, patients older than six years at the time of surgery, cases with negative ages due to birth year errors, and pediatric patients who had undergone cataract surgery before January 1, 2004 (S1 Fig). The overall study period was from January 2002 to December 2019, with at least an 8-year follow-up period to ensure consistent tracking of all patients. A washout period was set between January 1, 2002, and the date of the first cataract surgery to capture newly operated cataract patients. The intake period for the study was from January 2004 to December 2011, with the index date defined as the day following the first cataract surgery. The follow-up period was extended for 8 years after the index date (S2 Fig).

### Outcomes

#### Primary outcomes.

We defined the primary outcomes as healthcare resource utilization (HRU) and cataract-related medical costs in eligible pediatric patients with cataract who underwent cataract surgery over an 8-year follow-up period. HRU encompassed outpatient visits, inpatient admissions, and emergency department visits. Medical costs were calculated based on claims related to medications, surgeries, medical supplies, and medical procedures performed by doctors. The total medical costs were determined by summing the costs associated with each category. The median medical cost per patient over the 8 years was calculated. Additionally, we analyzed the medical costs for patients who developed severe postoperative complications, specifically retinal detachment or glaucoma, at least once during the 8-year follow-up.

We also derived annual HRU and medical costs during the follow-up period. We presented outcomes for each year, from the first to the eighth year following cataract surgery. Only the HRU and costs incurred at the ophthalmology department were considered cataract-related outcomes, and costs of cataract surgery performed prior to the index date were not included in the analysis. We used the 2022 currency exchange rate for calculations (1 USD = 1,292.2 KRW).

#### Secondary outcomes.

The secondary outcomes of this study were factors influencing medical costs following pediatric cataract surgery. The dependent variable was the 8-year cataract-related costs after cataract surgery. Independent variables included demographics (age, sex, income level, residence, and type of medical institution) and surgery-related factors, such as the type of cataract surgery, presence of complications (retinal detachment, glaucoma), and frequency of cataract surgeries.

### Subgroup analysis

A subgroup analysis was conducted to determine the additional disease burden in patients who developed ophthalmic complications after cataract surgery. Complications such as glaucoma, endophthalmitis, strabismus, amblyopia, and retinal detachment can occur postoperatively; however, this study specifically focused on “retinal detachment” and “glaucoma” owing to their commonality, severity, and the frequent necessity for surgical intervention in pediatric patients with cataract [6,19]. We calculated the frequency of complications, the time to the first complication after surgery, and complication-related medical costs in these patients. The index date for the analysis of this subgroup was set as the date of the postoperative complication diagnosis, with a median follow-up duration of 6.7 years; the longest obtainable period, as complications typically occurred within 1.3 years of surgery.

### Operational definitions

In this study, the HRU was categorized into outpatient visits, inpatient admissions, and emergency department visits. Outpatient visits and inpatient admissions were identified using specific form codes (outpatient: 03, 08; inpatient admission: 02, 07), excluding cases with emergency procedure codes. Emergency department visits were defined as visits charged to the emergency department or those with a corresponding emergency procedure code but not outpatient or inpatient form codes. Cataract surgery was identified using procedure codes S5110, S5111, S5112, or S5119. Regarding complications following cataract surgery, retinal detachment was defined using the procedure codes S5130 (scleral buckling), S5121 (total vitrectomy), or S5122 (partial vitrectomy), and the diagnosis code H33 (retinal detachment from the International Classification of Disease 10th revision code), whereas glaucoma was defined using the intraocular pressure lowering procedures coded as S5040-S5049. These operational definitions were verified by an ophthalmologist (S1 Table). Additionally, as the NHIS database provides only the birth year, the age of the patients was calculated as “year of first cataract surgery - year of birth”.

### Statistical analysis

Results were reported as medians with interquartile ranges (Q1, Q3) and arithmetic means with standard deviations (SD). A *p*-value < 0.05 was considered statistically significant.

To identify factors influencing medical costs after pediatric cataract surgery, we used generalized linear models (GLM), which are suitable for modeling skewed, non-negative continuous data such as healthcare costs [20]. Given the right-skewed distribution and the assumption that the variance increases proportionally with the mean, we specified a gamma distribution for the error structure. A log link function was used to ensure positive predicted outcomes and account for the multiplicative relationship [20,21]. Coefficients were presented in their exponential form.

Statistical analyses were conducted using SAS version 9.4 (SAS Institute Inc., Cary, NC, USA). Institutional Review Board (IRB)/Ethics Committee approval was obtained from Seoul National University Bundang Hospital (Approval No. X-2203-744-901), and the study was conducted according to the guidelines of the Declaration of Helsinki. This study utilized pseudonymized claims data provided by the NHIS and did not include any directly identifiable personal information. All handling of the data followed IRB-approved protocols.

## Results

After applying the eligibility criteria, we identified 1,297 pediatric patients aged 0–5 years who underwent their first cataract surgery within the study period. The mean age was 2.18 years, with the highest proportions in the 0 and 1-year-old groups (23.2% and 22.5%, respectively), and the majority were male (56.8%). Regarding socioeconomic status, the largest segment of patients (37.2%) was within the 11th to 15th income quartiles (level 3). Regarding surgery types, 60.9% underwent both extra- and intra-lens extraction. After the cataract surgery, 23 patients experienced retinal detachment, and 36 developed glaucoma requiring further surgery (Table 1).

**Table 1:** Baseline characteristics of the study population.

Characteristic		No. (n = 1,297)	%
Gender	Male	737	56.82
	Female	560	43.18
Mean age (mean, SD)		2.18	1.79
Age^a^	0 year old	301	23.21
	1 year old	292	22.51
	2 years old	159	12.26
	3 years old	158	12.18
	4 years old	192	14.8
	5 years old	195	15.03
Residence type	Seoul (Capital city)	371	28.6
	Metropolitan city	276	21.28
	Others	632	48.73
Income level^b^	level 0	9	0.69
	level 1	158	12.18
	level 2	353	27.22
	level 3	483	37.24
	level 4	259	19.97
Insurance type	Medicaid	1,288	99.31
	Commercial	9	0.69
Surgery type	Pars Plana Lensectomy	27	2.08
	Extracapsular of intracapsular extraction	790	60.91
	Surgery for after cataract	16	1.23
	Phacoemulsification	464	35.77
Hospital type	General Hospital	1,261	97.22
	Hospital	28	2.16
	Clinic	8	0.62
Disability level	Level 1	14	1.08
	Level 2	3	0.23
	Level 6	6	0.46
	None	1,274	98.23
Disability type	Ophthalmic disability	16	1.23
	Other disabilities^c^	7	0.54
	None	1,274	98.23
Complication type	Retinal detachment	23	1.77
	Glaucoma	36	2.78

^a^Age at cataract surgery is calculated as “year of surgery - year of birth” because the NHIS claims database only contains birth year data.

^b^Income level is categorized into quintiles, with level 0 representing the 0th quintile, level 1 representing the 1st-5th quintile, level 2 representing the 6th-10th quintile, level 3 representing the 11th-15th quintile, and level 4 representing the 16th-20th quintile; within the NHIS program, a higher income level number indicates a higher income.

^c^Cerebral lesion disorder, mental retardation, and intellectual disability were included.

NHIS, National Health Insurance Service; SD, standard deviation.

Table 2 presents the HRU findings and cataract-related medical costs in the ophthalmology department over an 8-year follow-up period for pediatric patients who underwent cataract surgery. On average, each patient had 30 outpatient visits, 2.7 inpatient admissions, and 3 emergency department visits after the surgery. The respective median costs per patient in these settings were $661 for outpatient visits, $1,346 for inpatient admissions, and $122 for emergency department visits. Additionally, patients who developed retinal detachment following cataract surgery incurred a median 8-year cost of $4,347. Those with glaucoma incurred a median cost of $6,754, predominantly in the inpatient setting (S2 Table**).**

**Table 2:** Healthcare resource utilization and medical costs* over 8 years after cataract surgery in pediatric cataract patients.

No. of patients	N	
Patients with cataract	1,297	
Patients with retinal detachment after cataract surgery	23	
Patients with glaucoma after cataract surgery	36	
	**Median (Q1-Q3)**	**Mean**^a^ **(SD)**
**Health resource utilization for 8 years (per patient)**		
No. of outpatient visits^b^	26 (19-37)	30.08 (18.08)
No. of inpatient visits^c^	2 (1-4)	2.69 (2.29)
No. of emergency department visits^d^	2 (1-3)	3.03 (3.45)
**Medical costs associated with cataracts for 8 years (per patient)**		
Total medical costs	$1,728 (763−3,033)	$2,391 (3,404)
In outpatient setting	$661 (431-943)	$750 (508)
In inpatient setting	$1,346 (720−2,667)	$2,171 (3,600)
In an emergency department setting	$122 (59-251)	$217 (349)
Patients with retinal detachment after cataract surgery	$4,347 (2,631−11,496)	$7,729 (6,960)
Patients with glaucoma after cataract surgery	$6,754 (4,226−10,024)	$8,821 (9,422)

* Health resource utilization records and costs generated by the ophthalmology department were included, and cataract surgery costs were not included. The mean value is the sum of the costs derived over the cost analysis period (8 years) divided by the number of subjects in each disease category, excluding the cost of the cataract surgery.

^a^The mean was calculated by dividing total costs by the number of patients. This calculation method was also applied to determine the mean HRU per capita.

^b^Outpatient visits were identified based on claims from outpatient visits through medical institutes without emergency department claims.

^c^Inpatient admissions were identified based on claims from inpatient visits through medical institutes without emergency department claims.

^d^Emergency department visits were identified based on emergency claims with an emergency department visit.

Q1, first quartile; Q3, third quartile; SD, standard deviation; $, United States Dollar.

**Fig 1:**
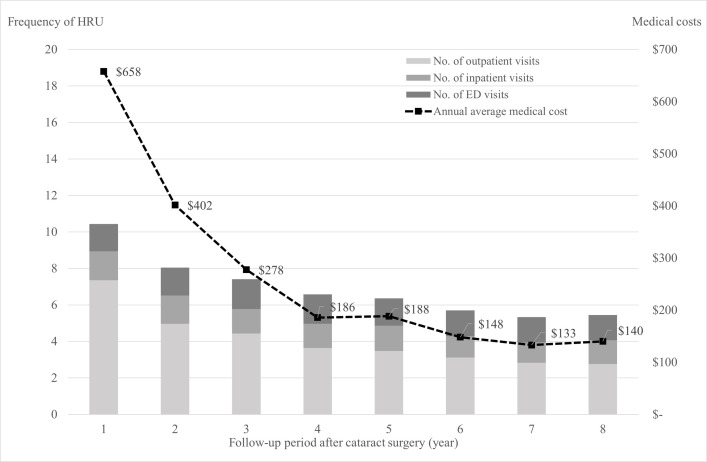
Trends in annual medical costs and healthcare resource utilization (HRU) and per patient over an 8-year follow-up period after pediatric cataract surgery. The bar chart represents average annual frequencies of outpatient, inpatient, and emergency department (ED) visits, while the dashed line shows average annual medical costs per patient (in USD) among 1,297 patients.

Fig 1 illustrates the trends in annual HRU and medical costs per patient over 8-year follow-up after pediatric cataract surgery. Medical costs dropped sharply from $658 in Year 1 to $402 in Year 2, followed by a gradual decline over time. The average medical costs were $1,337 in the short term (Years 1–3), $522 in the medium term (Years 4–6), and $273 in the long term (Years 7–8). Similarly, the average number of annual HRU visits decreased from approximately 10 visits in Year to 5 visits by Year 8. Overall, both medical costs and HRU showed a consistent decrease over time.

The results of the GLM analysis identifying factors influencing medical costs over 8 years following cataract surgery are presented in **Table 3**. The analysis revealed that older age at surgery correlated with higher medical costs. Patients with higher income levels incurred 0.4–0.5 times more expenses compared to those at the lowest income level. Notably, patients who experienced retinal detachment and glaucoma after cataract surgery incurred significantly higher costs (3.4 and 3.7 times more, respectively), than those without complications. Additionally, an increased number of cataract surgeries led to 1.7 times higher costs over 8 years. Factors such as sex, residence, medical institution type, and surgery type did not significantly impact the costs.

**Table 3:** Generalized linear model analysis of factors affecting 8-year medical costs following cataract surgery (n = 1,297).

Parameter	Beta^a^	SE	95% CI		*p*-value
**Intercept**	3,660,000	0.31	2,010,000	6,670,000	<.0001
**Demographic factors**					
** Age**	0.88	0.01	0.86	0.90	<.0001
** Female gender** *(ref: male)*	0.97	0.04	0.89	1.06	0.55
** Residence** *(ref: capital city)*					
** Metropolitan city**	0.97	0.06	0.86	1.09	0.58
** Others**	1.05	0.05	0.95	1.16	0.32
**Health institute** *(ref: general hospital)*					
** Hospital**	1.03	0.15	0.77	1.38	0.84
** Clinic**	0.92	0.27	0.54	1.58	0.77
**Income level**^b^ *(ref: Level 0)*					
** Level 1**	0.45	0.27	0.75	0.27	<.05
** Level 2**	0.52	0.31	0.88	0.26	<.05
** Level 3**	0.49	0.29	0.82	0.26	<.05
** Level 4**	0.41	0.25	0.69	0.27	<.05
**Surgery-related factors**					
** Cataract surgery type** *(ref: Pars Plana Lensectomy)*					
** Extracapsular of intracapsular extraction**	0.81	0.16	0.60	1.10	0.18
** Surgery for after cataract**	0.75	0.24	0.47	1.20	0.23
** Phacoemulsification**	0.97	0.16	0.71	1.32	0.86
** Complication type** *(ref: no complication)*					
** Retinal detachment**	3.35	0.16	2.43	4.61	<.0001
** Glaucoma**	3.70	0.13	2.86	4.76	<.0001
** Number of cataract surgery**	1.70	0.03	1.59	1.82	<.0001

^a^The exponentiated form of estimated coefficients indicates the multiplicative change in 8-year medical costs for a one-unit increase in the predictor.

^b^In the NHIS program, a higher income level indicates a higher income.

CI, confidence interval; SE, standard error.

Among patients who developed complications after cataract surgery, retinal detachment (n = 21) was observed 1.5 times, on average, over 6.7 years, with the first incidence typically around 5.5 years after surgery, leading to a median additional medical cost of $4,616 per patient. Similarly, glaucoma (n = 40) occurred approximately twice on average, with the first episode occurring approximately 5.2 years after surgery, resulting in a median additional cost of $3,617 per patient (**Table 4**).

**Table 4:** Complication frequency, duration, and medical costs over 6.7 years among patients with complications after cataract surgery.

	Retinal detachment (n = 21)	Glaucoma (n = 40)
**Number of complications**		
** **Mean (SD)	1.48 (0.6)	1.98 (1.42)
**Duration of 1**^**st**^ **complication between cataract surgery (day)**		
** **Median (Q1-Q3)	2,226 (228−3,833)	1,465 (651−2,948)
** **Mean (SD)	2,030 (1,745)	1,906 (1,433)
**Medical costs associated with complications per patient**		
** **Median (Q1-Q3)	$4,616 (2,089−5,861)	$3,617 (2,338−5,168)
** **Mean (SD)	$4,705 (3,116)	$4,608 (3,532)

Q1, 1^st^ quartile; Q3, 3^rd^ quartile; SD, standard deviation; $, United States Dollars.

## Discussion

We examined the ophthalmic complications and economic burden following cataract surgery among pediatric patients, and factors influencing their medical costs in South Korea. The results demonstrated that pediatric cataract surgery had a significant impact on long-term economic burden and HRU. The age at surgery, income level, occurrence of complications, and number of cataract surgeries were factors associated with increased economic burden. The association between older age at surgery and higher medical costs may reflect increased clinical complexity and a greater need for follow-up care in older pediatric patients. Similarly, higher income levels were also associated with increased costs, which may be explained by greater healthcare access, use of additional services, and potentially higher expectations for follow-up care among higher-income families. Furthermore, by analyzing the additional medical expenses associated with complications following cataract surgery, we found that patients who experienced complications had additional burdens besides cataracts.

Previous studies on the cost of pediatric cataracts have predominantly utilized hospital data. Evans et al. reported annual direct costs related to pediatric cataract surgery in Zambia and Malawi (n = 232), ranging from $202 to $277 per person [12]. Similarly, Wang et al. estimated the total medical burden for patients under 10 years of age in China (n = 181) at $3,850, covering the pre-surgery, surgery, and post-surgery periods [11]. However, neither study adopted a specific follow-up period, preventing a precise assessment of postoperative cataract-related medical costs. Additionally, the cost data reported by Wang et al. were derived from medical receipts collected via questionnaires, possibly resulting in incomplete data and unreliable results. Both studies have limitations in representativeness owing to data confinement to specific regional hospitals, and institutional disparities between medical facilities further challenge the generalization of their findings [11,12].

In contrast, the Infant Aphakia Treatment Study, a clinical trial conducted in the United States, evaluated the 5-year treatment costs for infants with cataracts (n = 144) based on different vision correction methods. This study included the cost of consumables such as contact lenses and glasses, which are non-reimbursable in Korea, leading to a higher cost estimate than ours. Excluding these consumables, the 5-year cost ranged from $6,766 to $7,509 [22].

In South Korea, most studies on pediatric patients with cataracts have been limited to case reports that share clinical outcomes from single hospitals or compare surgical techniques [7,23–25]. Although these studies provide valuable clinical insights, they do not extensively address the economic aspects of pediatric cataract treatment. Ryu et al. estimated the total annual cost of cataract surgery at approximately 400 billion KRW, using data from the NHIS. However, their study primarily focused on senile cataracts of the elderly population with an average age of 69 years, and it did not provide specific insights into the pediatric population. It lacked details such as the median or average cost per pediatric patient and the pattern of medical expenses, which are crucial for understanding the financial burden of pediatric cataract surgery [16]. As virtually nothing is known about the economic burden of pediatric patients suffering cataracts, our results addressing HRU, medical costs, and its determinants are notable.

The clinical care pathway for pediatric cataract varies across countries, influenced by socioeconomic status, healthcare infrastructure, and resource availability. In high-income countries, timely surgical intervention and comprehensive postoperative care are generally more accessible, leading to better visual outcomes. In contrast, low- and middle-income countries often face delays in diagnosis and treatment due to limited healthcare access, financial constraints, and geographic barriers [26,27]. South Korea’s healthcare system, characterized by universal health coverage and high accessibility, enables early surgical intervention and consistent follow-up care. The incidence of pediatric cataract surgery in Korea is comparable to other high-income countries [16]. Therefore, while the absolute healthcare costs may differ due to variations in reimbursement systems (e.g., private insurance coverage for premium intraocular lenses or non-reimbursed services), the economic burden patterns identified in this study are likely generalizable to other high-income settings with similarly structured healthcare systems [28].

Several Asian countries with universal health insurance and high-quality ophthalmic infrastructure, such as Japan and Taiwan, provide a useful comparative context. For example, Taiwan has demonstrated that shifts in reimbursement policies (e.g., from fee-for-service to case payment) influence the adoption of advanced surgical techniques and long-term outcomes. Japan introduced the Diagnosis Procedure Combination (DPC) system, a per diem prospective payment system, to reduce hospital stays and control medical costs. The DPC system can reduce medical costs for cataract operations, which generally involve a longer length of stay [29,30]. These examples suggest that cost-control policies and reimbursement models significantly shape care pathways and economic burdens. Given the structural similarities of these healthcare systems to those of South Korea, our findings may inform broader policy discussions. Specifically, they support the need to promote equitable access to premium intraocular lenses (e.g., through modified reimbursement criteria), establish standardized postoperative monitoring protocols to minimize complication-related expenditures, and evaluate the feasibility of bundled or case-based payment models tailored to pediatric cataract care.

The strength of this study lies in its detailed and comprehensive analysis of medical costs and HRU in pediatric patients with cataracts using long-term, nationwide, population-based data. First, unlike most cataract research, which focused on adult populations, our study provides representative, real-world evidence of the economic burden associated with pediatric cataracts by utilizing data from a nationwide database. Given the rarity of pediatric cataracts, the comprehensive analysis of a population of 50 million ensures strong representativeness, minimizing the risk of missing data and reducing selection bias, which are significant strengths of this study. Second, the extended follow-up period of 8 years enabled us to meticulously track and analyze long-term trends in medical costs and resource utilization, offering invaluable insights into the prolonged economic impact of pediatric cataract surgery. Lastly, a notable methodological advancement in this study was the application of a GLM to address the skewed distribution typically observed in cost data. This approach allowed for a more precise analysis of the factors influencing medical costs. By correcting this skewness, our study could deliver a more accurate understanding of the economic burden associated with pediatric cataract surgery, thereby significantly enhancing the robustness of our findings.

Nonetheless, this study had several limitations, primarily owing to its retrospective nature and the data utilized. First, this study used data from 2004 to 2011 and thus does not reflect changes in surgical techniques or reimbursement policies. Since July 2012, cataract surgery has been included in the Diagnosis Related Group payment system, which may have reduced inpatient reimbursement levels and altered the cost structure. Advances in surgical techniques and a shift toward outpatient-based care may also have contributed to lower per-case costs. While the NHIS statistical yearbook suggests that per-person costs have remained relatively stable since 2012 (S3 Fig), future studies using updated data are needed to capture recent trends. Second, this study relied on claims data, which did not include non-reimbursement items such as therapeutic contact lenses, glasses used for visual rehabilitation, or biometry tests. This omission may result in a conservative estimation of medical costs and may not fully capture the true economic burden of pediatric cataract surgeries [19,31,32]. Similarly, the NHIS claims data do not include quality-of-life or functional vision outcomes, which limits assessing the broader clinical impact of pediatric cataract. Future studies incorporating clinical or patient-reported data are needed to address this gap. Furthermore, the retrospective nature of the data may also limit external validity, and findings should be interpreted with caution. Third, glaucoma was defined solely based on surgical codes, excluding patients treated with medication alone, which resulted in a small number of patients identified as having complications. This likely led to an underestimation of the total glaucoma population and its associated costs. Nevertheless, by focusing on surgically treated cases, our analysis reflects patients with more severe status and thus provides a conservative estimate of the complication-related cost burden. Fourth, formal goodness-of-fit diagnostics for the GLM (e.g., residual analysis, overdispersion checks) were not performed, which may limit the ability to fully validate model assumptions. However, the selected model structure is consistent with standard approaches for skewed cost data and was deemed appropriate based on prior evidence. Finally, we did not include costs for post-surgical visual rehabilitation tools, such as therapeutic lenses or glasses, as these are typically non-reimbursed and not captured in claims data. This might lead to an underestimation of the real-world economic burden on patients and caregivers, suggesting that actual cataract-related costs may be higher than reported.

Despite the mentioned limitations, we believe this study generated robust, long-term cost estimates for pediatric cataract patients, a population for which economic data is scarce. Unlike previous studies that focused on adult populations or small sample sizes, our research provides rare, population-level evidence in a vulnerable pediatric group by utilizing a large, nationally representative claims database.

The findings have practical implications for healthcare decision-makers. By identifying high-cost drivers such as postoperative complications and repeated surgeries, this study highlights the importance of early intervention and sustained long-term care. These insights can support more effective preventive strategies, resource prioritization, and healthcare planning in the field of pediatric ophthalmology.

In conclusion, we estimated long-term medical costs and key contributing factors—such as age, income level, and complications—among pediatric patients undergoing cataract surgery. These results provide meaningful economic evidence that can inform future policy and improve care strategies for children at risk of visual impairment.

## Supporting information

S1 Fig Patient identification and selection process. (TIF)

S2 Fig Analysis scheme of the retrospective longitudinal study design. (TIF)

S3 Fig Annual trend of the number of patients and medical costs per patient per year for children under 5 years old who underwent cataract surgery. Patients under 5 years old who underwent cataract surgery (procedure codes: S5110, S5111, S5112, S5119) from 2010 to 2019 were included. Data source: Open statistics of procedure codes from the Health Insurance Review and Assessment Service. Accessed from: https://opendata.hira.or.kr/op/opc/olapDiagBhvInfoTab2.do?moveFlag=Y
(TIF)

S1 Table Codes of operational definitions. (DOCX)

S2 Table Medical costs over 8 years among patients with complications after cataract surgery. (DOCX)
