# Supplementary figures and images for: Long-term economc burden and related influencing factors of pediatric cataracst: A population-based study in South Korea
Source: PLoS One. 2025 Aug 21;20(8):e0328781. doi: 10.1371/journal.pone.0328781 (PMC12370042; doi:10.1371/journal.pone.0328781)

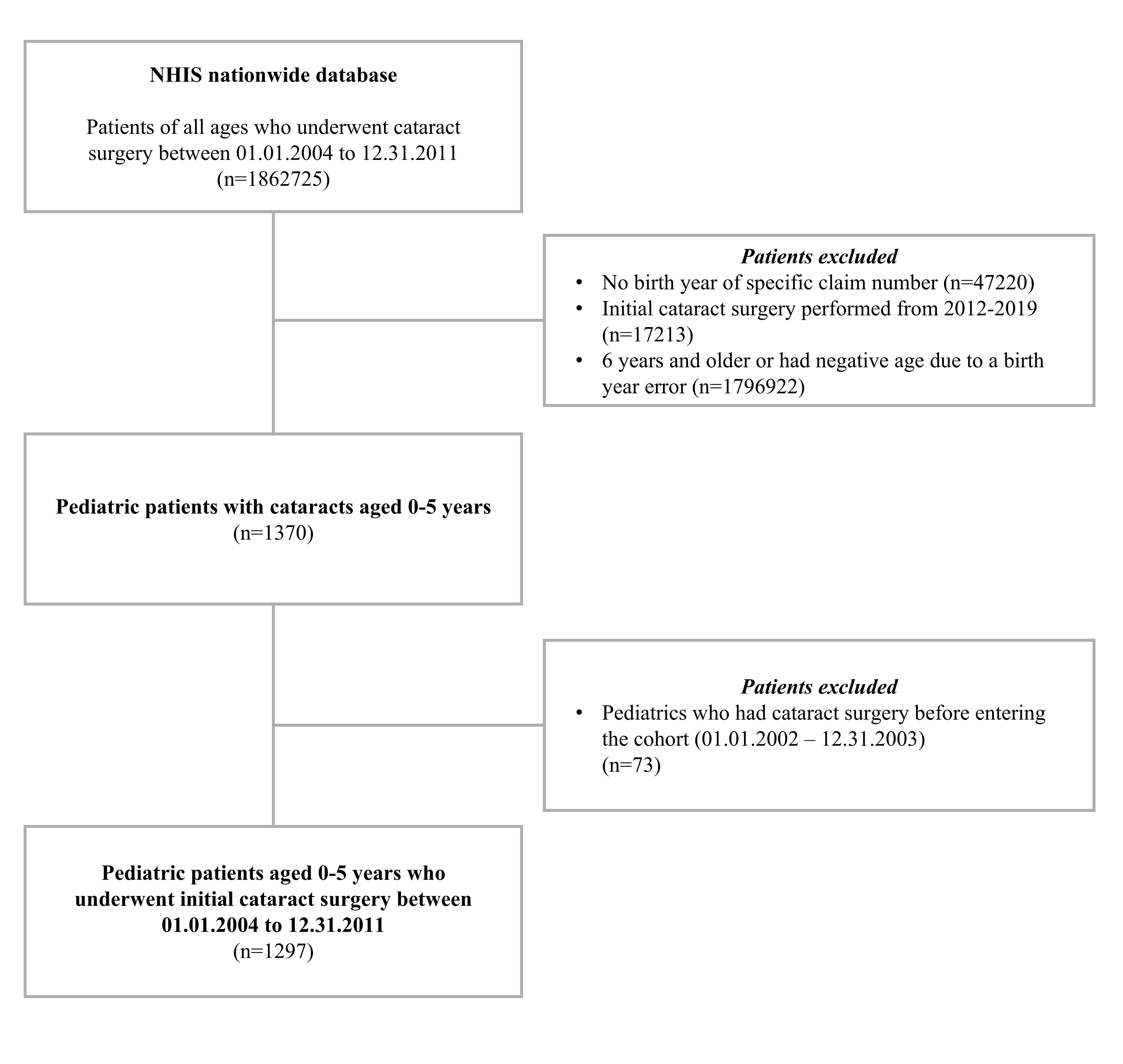

Supplement: S1 Fig — (TIF) [file pone.0328781.s001.tif]

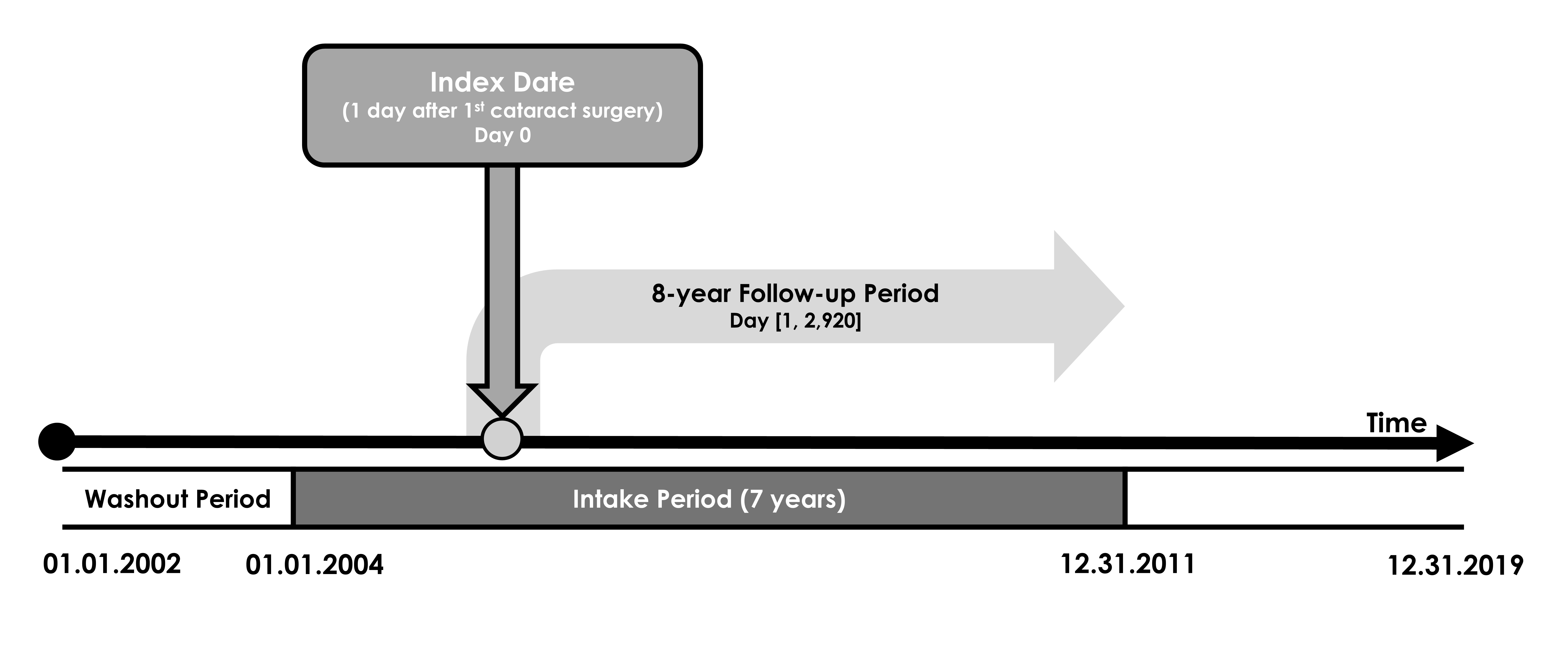

Supplement: S2 Fig — (TIF) [file pone.0328781.s002.tif]

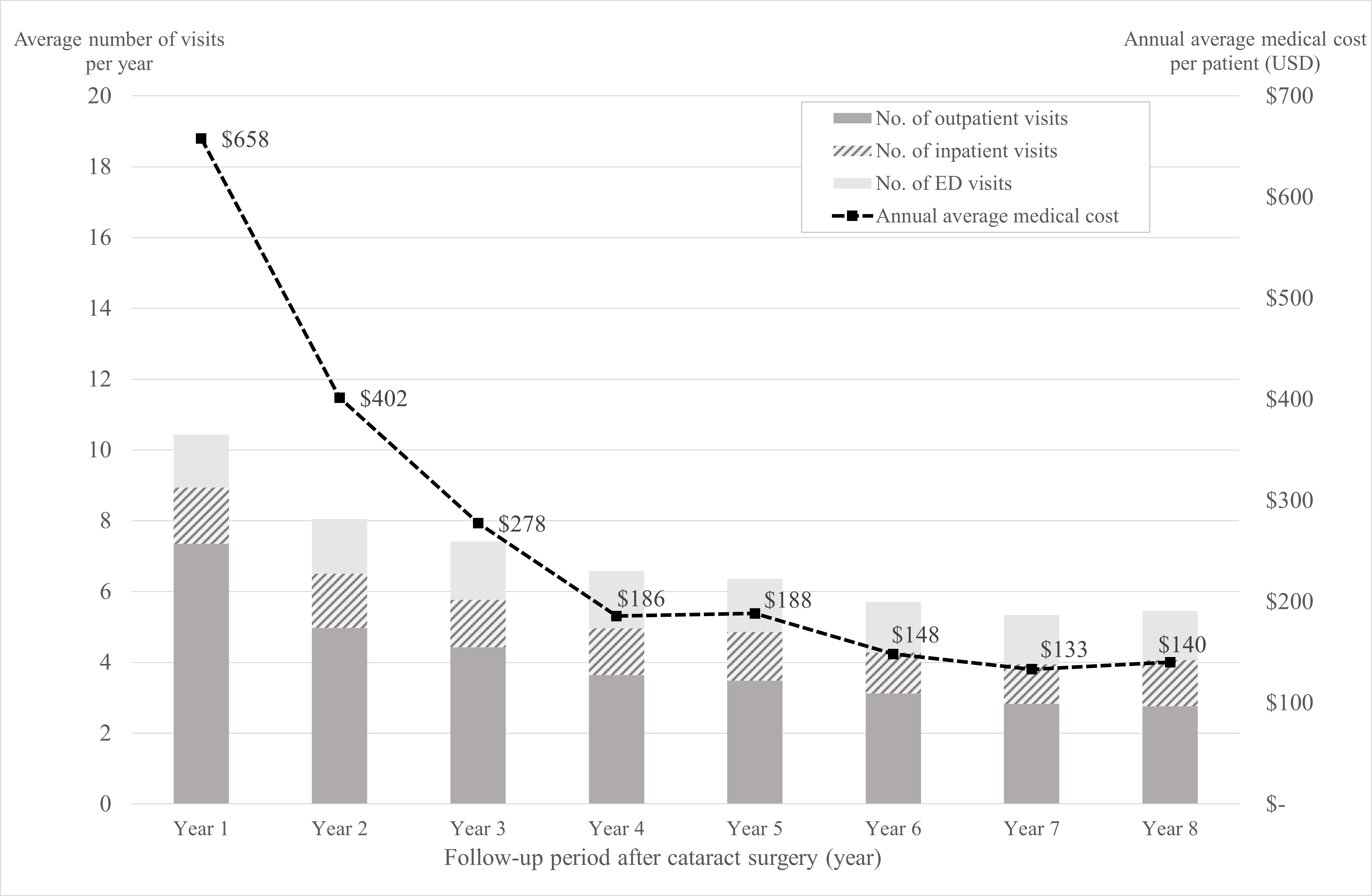

Supplement: S3 Fig — Patients under 5 years old who underwent cataract surgery (procedure codes: S5110, S5111, S5112, S5119) from 2010 to 2019 were included. Data source: Open statistics of procedure codes from the Health Insurance Review and Assessment Service. Accessed from: https://opendata.hira.or.kr/op/opc/olapDiagBhvInfoTab2.do?moveFlag=Y (TIF) [file pone.0328781.s003.tif]
