# Supplementary material for: Long-term economc burden and related influencing factors of pediatric cataracst: A population-based study in South Korea
Source: PLoS One. 2025 Aug 21;20(8):e0328781. doi: 10.1371/journal.pone.0328781 (PMC12370042; doi:10.1371/journal.pone.0328781)
Supplement: S1 Table — (DOCX) [file pone.0328781.s004.docx]

S1 Table. Codes of operational definitions

| **Code** | **Name** | **Type of code** |
| --- | --- | --- |
| ***Health resource utilization*** | | |
| **Outpatient visit** | | |
| 03 | Hospital Outpatient | Claims form code |
| 08 | Clinic Outpatient |  |
| **Inpatient hospitalization** | | |
| 02 | Hospital Inpatient | Claims form code |
| 07 | Clinic Inpatient |  |
| **Emergency department visit** | | |
| V1100 | Emergency Medical Management Fee - Central Emergency Medical Center | Procedure code |
| V1200 | Emergency Medical Management Fee - Regional Emergency Medical Center (Class B) |  |
| V1210 | Emergency Medical Management Fee - Regional Emergency Medical Center (Class A) |  |
| V1220 | Emergency Medical Management Fee - Regional Emergency Medical Center (Class C) |  |
| V1300 | Emergency Medical Management Fee - Local Emergency Medical Center (Class B) |  |
| V1310 | Emergency Medical Management Fee - Local Emergency Medical Center (Class A) |  |
| V1320 | Emergency Medical Management Fee - Local Emergency Medical Center (Class C) |  |
| V1400 | Emergency Medical Management Fee - Local Emergency Medical Services Agency |  |
| ***Cataract surgery*** | | |
| S5110 | Pars Plana Lensectomy | Procedure code |
| S5111 | Extracapsular of intracapsular extraction |  |
| S5112 | Surgery for after cataract |  |
| S5119 | Phacoemulsification |  |
| ***Complications*** | | |
| **Retinal detachment** | | |
| S5121 | Vitrectomy-Total | Procedure code |
| S5122 | Vitrectomy-Partial |  |
| S5130 | Retinal Detachment Surgery |  |
| H33 | Retinal detachments and breaks | ICD-10 disease code |
| **Glaucoma** | | |
| S5040 | Surgery for Glaucoma-Nonpenetrating Filtration Surgery (Deep Sclerectomy, Viscocanalostomy) | Procedure code |
| S5041 | Surgery for Glaucoma-Iridectomy |  |
| S5042 | Surgery for Glaucoma-Filtering Operation |  |
| S5043 | Surgery for Glaucoma-Trabeculectomy |  |
| S5044 | Surgery for Glaucoma-Photocoagulation for Iris, Ciliary Body |  |
| S5045 | Surgery for Glaucoma-Cyclocryotherapy |  |
| S5047 | Surgery for Glaucoma-Trabeculotomy Under Microscopy |  |
| S5048 | Surgery for Glaucoma-Sinusotomy Under Microscopy |  |
| S5049 | Glaucoma Implant Surgery |  |
| Abbreviations: ICD-10, International Classification of Disease 10^th^ revision. | | |
