# Supplementary material for: Long-term economc burden and related influencing factors of pediatric cataracst: A population-based study in South Korea
Source: PLoS One. 2025 Aug 21;20(8):e0328781. doi: 10.1371/journal.pone.0328781 (PMC12370042; doi:10.1371/journal.pone.0328781)
Supplement: S2 Table — (DOCX) [file pone.0328781.s005.docx]

S2 Table. Medical costs over 8 years among patients with complications after cataract surgery

|  | **Median (Q1-Q3)** | **Mean (SD)** |
| --- | --- | --- |
| **Medical costs for retinal detachment for 8 years (per patient)** | | |
| No. of patient |  | 23 |
| Total medical costs | $4347 (2631-11496) | $7729 (6960) |
| In outpatient setting | $784 (393-1084) | $902 (726) |
| In inpatient setting | $3802 (2046-11440) | $6583 (6209) |
| In an emergency department setting | $128 (33-575) | $374 (585) |
| **Medical costs associated with glaucoma for 8 years (per patient)** | | |
| No. of patient |  | 36 |
| Total medical costs | $6754 (4226-10024) | $8821 (9422) |
| In outpatient setting | $1561 (1180-2429) | $1848 (1036) |
| In inpatient setting | $5133 (2507-7227) | $6849 (8872) |
| In an emergency department setting | $100 (60-168) | $155 (148) |
| Abbreviations. Q1, first quartile; Q3, third quartile; SD, standard deviation. | | |
|  | | |
